# Fucoxanthin and Colorectal Cancer Prevention

**DOI:** 10.3390/cancers13102379

**Published:** 2021-05-14

**Authors:** Masaru Terasaki, Atsuhito Kubota, Hiroyuki Kojima, Hayato Maeda, Kazuo Miyashita, Chikara Kawagoe, Michihiro Mutoh, Takuji Tanaka

**Affiliations:** 1 School of Pharmaceutical Sciences, Health Sciences University of Hokkaido, 1757 Kanazawa, Ishikari-Tobetsu, Hokkaido 061-0293, Japan; atsuhito_k@hoku-iryo-u.ac.jp (A.K.); hirokojima@hoku-iryo-u.ac.jp (H.K.); 2 Advanced Research Promotion Center, Health Sciences University of Hokkaido, 1757 Kanazawa, Ishikari-Tobetsu, Hokkaido 061-0293, Japan; 3 Faculty of Agriculture and Life Science, Hirosaki University, 3 Bunkyo-cho, Hirosaki, Aomori 036-8561, Japan; hayatosp@hirosaki-u.ac.jp; 4 Center for Industry-University Collaboration, Obihiro University of Agriculture and Veterinary Medicine, Inada-cho, Obihiro, Hokkaido 080-8555, Japan; miyashitak@obihiro.ac.jp; 5 Algatech Kyowa, Kyowa Concrete Industry Co. Ltd., Hakodate, Hokkaido 040-0051, Japan; kawagoe.c@kyowa-concrete.co.jp; 6 Department of Molecular-Targeting Prevention, Graduate School of Medical Science, Kyoto Prefectural University of Medicine, Kawaramachi-Hirokoji, Kamigyo-ku, Kyoto 602-856, Japan; mimutoh@koto.kpu-m.ac.jp; 7 Department of Diagnostic Pathology and Research Center of Diagnostic Pathology, Gifu Municipal Hospital, 7-1 Kashima-cho, Gifu 500-8513, Japan; takutt@gmhosp.gifu.gifu.jp

**Keywords:** fucoxanthin, colorectal cancer, cancer prevention, carotenoid, tumor microenvironment, gut microbiota

## Abstract

**Simple Summary:**

Colorectal cancer (CRC) is suggested to be preventable by certain food intakes. Fucoxanthin (Fx) is an anticancer agent contained abundantly in edible brown algae. However, epidemiological studies, in vivo and in vitro experiments for CRC, using Fx and Fx-rich foods, have not been fully outlined. To date, it has been reported that Fx, its metabolite of fucoxanthinol (FxOH) and Fx-rich algal extracts exerted anticancer potentials in human CRC cell lines, their cancer stem-cells-like spheroids and CRC animal models through a number of molecular mechanisms. Moreover, many in vivo experiments and interventional human trials have demonstrated that Fx, Fx-rich algal extracts and brown alga itself may improve CRC and/or certain risks, such as obesity, diabetes, metabolic syndrome, inflammation, oxidation, tumor microenvironment and/or gut microbiota. This review is the first report that summarizes the improving effects by Fx, FxOH and its rich brown algae for CRC and the risk factors.

**Abstract:**

Colorectal cancer (CRC), which ranks among the top 10 most prevalent cancers, can obtain a good outcome with appropriate surgery and/or chemotherapy. However, the global numbers of both new cancer cases and death from CRC are expected to increase up to 2030. Diet-induced lifestyle modification is suggested to be effective in reducing the risk of human CRC; therefore, interventional studies using diets or diet-derived compounds have been conducted to explore the prevention of CRC. Fucoxanthin (Fx), a dietary carotenoid, is predominantly contained in edible brown algae, such as *Undaria pinnatifida* (wakame) and *Himanthalia elongata* (Sea spaghetti), which are consumed particularly frequently in Asian countries but also in some Western countries. Fx is responsible for a majority of the anticancer effects exerted by the lipophilic bioactive compounds in those algae. Interventional human trials have shown that Fx and brown algae mitigate certain risk factors for CRC; however, the direct mechanisms underlying the anti-CRC properties of Fx remain elusive. Fx and its deacetylated type “fucoxanthinol” (FxOH) have been reported to exert potential anticancer effects in preclinical cancer models through the suppression of many cancer-related signal pathways and the tumor microenvironment or alteration of the gut microbiota. We herein review the most recent studies on Fx as a potential candidate drug for CRC prevention.

## 1. Introduction

Colorectal cancer (CRC) is a major global cancer, accounting for about 6% of total cases of new cancer (1.1 million per 18.1 million) and cancer death (0.6 million per 9.6 million), as estimated by the GLOBOCAN 2018 database [1]. The incidence and mortality of CRC have been declining in highly developed countries, such as the USA, Australia, Russia and Japan, where an early diagnosis, surgical resection and drug treatments are easy to receive. However, both the global incidence and mortality of CRC are expected to increase by 2030 due to an increasing trend in the CRC burden in many other countries [2]. 

The diagnosed types of CRC typically include CRC derived from polyp and inflammatory bowel disease (IBD)-derived CRC, with a low incidence of hereditable CRCs, such as Lynch syndrome (about 2–4%), familial adenomatous polyposis (FAP, about 1%), Peutz–Jeghers syndrome and MUTYH-associated polyposis [3]. The 5-year survival rate in CRC is 60–68% with racial diversity in all stages; the survival rate is about 90% when CRC is detected at an early stage before spreading [3]. Convincing and probable risk factors for CRC are as follows: IBD, Lynch syndrome, FAP, Peutz-Jeghers syndrome, MUTYH-associated polyposis, processed meat, alcoholic drinks, body fatness, adult attained height and red meat [4,5,6,7,8]. The key triggers involved in human colorectal carcinogenesis are high frequencies of gene mutations (e.g., adenomatous polyposis coli (*APC*), Kirsten-ras (*KRAS*), and *TP53*) and gene fusions, aberrant expressions of mRNA, micro RNA and long non-coding RNA, alterations of splicing event, core signal transduction, DNA repair, extracellular matrix construction and metabolism, microsatellite instability, hypermethylation, copy number variation, immune dysregulation and gut microbiota alteration [9,10,11,12,13,14,15]. Pathological diagnoses have revealed that CRC arises through multistep carcinogenesis from dysplastic crypts to adenocarcinoma. Mutated *APC*, *KRAS*, and *TP53* are strongly associated with the malignant progression of CRC [16,17,18,19]. In addition, the formation of immunosuppressive tumor microenvironment (TME) is essential for the onset of adenocarcinoma in colorectal mucosal tissue. The colorectal TME is composed of colorectal cancer stem cells (CCSCs), cancer-associated fibroblasts (CAFs), many immune cells, including tumor-associated macrophages (TAMs) and dendritic cells (DCs), modified extracellular matrix (ECM), stromal collagen enhancement and abnormal neovessels [20,21,22]. 

**Figure 1:**
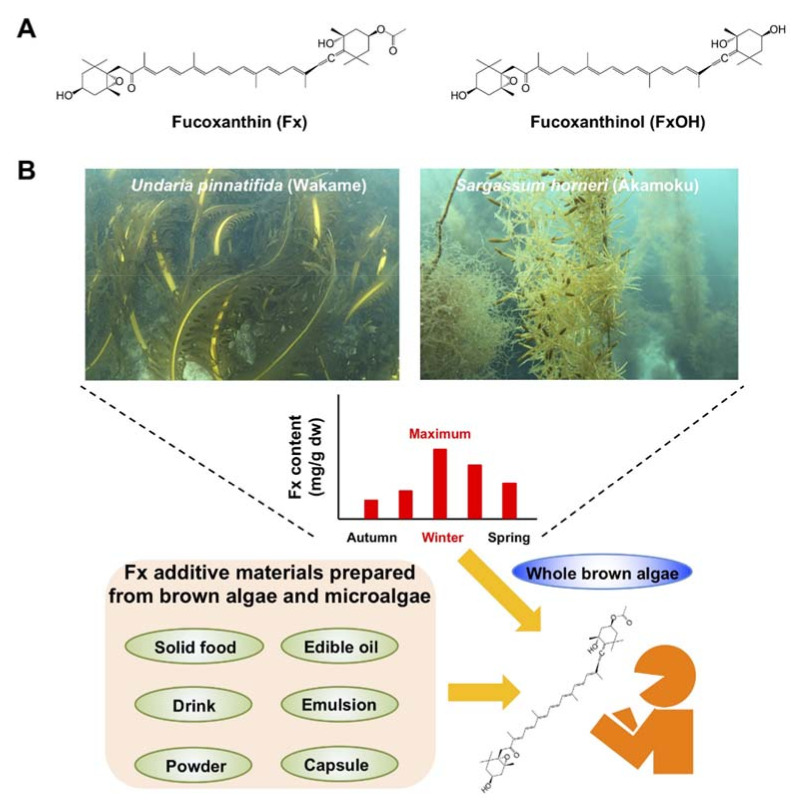
Ingestible routes of Fx in humans. (**A**) Chemical structures of fucoxanthin (Fx) and fucoxanthinol (FxOH). (**B**) Ingestible routes of Fx in humans. Whole brown algae, such as *Undaria pinnatifida* (Wakame) and *Sargassum horneri* (Akamoku), collected in the winter, when the content of Fx is the highest, may be beneficial for human health. Furthermore, Fx extracted from brown algae and microalgae can be added to various edible items to customize their route of ingestion by humans.

Fucoxanthin (Fx, Figure 1), a non-provitamin A carotenoid, is found abundantly in brown algae and microalgae. Fx binds the chlorophyll a/c-protein and contributes to efficient light harvesting for photosynthetic organisms as well as the body color. Fx has been estimated to account for >10% of total biogenic carotenoids [23]. It has an unusual allenic bond and a 5,6-monoepoxide, and its molecular weight is 658.9 g/mol (C_42_H_58_O_6_). Fx has been cleared as a safe carotenoid without any adverse effects at 0.5% (*w*/*v*) on human skin and at 20–2000 mg Fx/kg body weight (BW) in rodents [24,25,26]. Fx is easily metabolized to *cis*-Fx, fucoxanthinol (FxOH, Figure 1) in the intestine and then to amarouciaxanthin A (Amx A) and *cis*-Amx A in the liver. FxOH and *cis*-FxOH occur as the main plasma metabolites of human-ingested brown algae or its extract [27,28]. Hashimoto et al. showed that a single administration of an algal extract (31 mg Fx) resulted in a maximum concentration of 44.2 nmol/l, time at maximum concentration of 4.0 h and terminal half-life of 7.0 h for plasma FxOH [28]. In contrast, FxOH, Amx A and *cis*-Amx A are the predominant forms in the blood and various organs of mice administered Fx [29,30]. Several early studies have shown that Fx exerts important anti-inflammation [31], anti-obesity [32], anti-diabetes [33], anti-hypertension [34], anti-cardiovascular diseases [34], antimicrobial [35], antioxidation [36], photoprotection [37], anti-angiogenesis [38], anti-brain injury [39,40] and anticancer [41,42] effects in human, animal models and culture cells. 

Although many epidemiological studies have been conducted on nutritional approaches to CRC prevention, carotenoids—particularly non-polar carotenoids, such as α-carotene, β-carotene and lycopene—are still ranked as having limited evidence or no conclusion. However, carotenoids are abundantly contained in vegetables and fruits whose effects on CRC prevention are described as limited evidence to suggestive [8]. Furthermore, the effects of carotenoids vary widely depending on the polarity and include Fx, neoxanthin, violaxanthin, β-cryptoxanthin and lutein, α-carotene, β-carotene and lycopene. To date, epidemiological studies involving in vivo and in vitro experiments for CRC, using Fx itself and its rich foods, have not been fully outlined.

We herein review the most recent studies on Fx as a potential CRC preventive agent. There is little information available on a direct evidence of anti-CRC properties of Fx for patients; however, Fx and FxOH have been reported to exert potential anticancer effects in many CRC cell lines (summarized in Section 3), as well as in many preclinical CRC animal models (summarized in Section 4). In addition, many interventional human trials and in vivo studies have suggested that Fx and its rich brown algae may improve CRC and/or certain risks for CRC such as obesity, diabetes, metabolic syndrome, inflammation, oxidation, TME, and gut microbiota (summarized in Section 4 and Section 5). This is the first report integrated the improving effects by Fx and its rich brown algae for CRC and the risk factors.

## 2. Fx Sources in Foods and Other Materials

Brown algae, such as Heterokontophyta and Ochrophyta, are traditionally and most widely consumed in Asian countries, such as Japan, China and Korea [43]. However, brown algae are expanding steadily as novel foods in North America, South America and Europe [44]. The European market, in particular, is one of the most rapidly growing regions regarding the consumption of brown algae [45,46].

Four brown algae species—*Undaria pinnatifida* (Wakame), *Sargassum horneri* (Akamoku), *Hizikia fusiforme* (Hiziki) and *Saccharina japonica* (Makombu)—are habitually consumed as representative seafood sources in Asia. In Europe, nine species of edible brown algae—*Fucus vesiculosus*, *Fucus serratus*, *Himanthalia elongata* (Sea spaghetti), *U. pinnatifida*, *Ascophyllum nodosum*, *Laminaria digitate*, *Laminaria saccharina*, *Saccharina japonica* and *Alaria esculenta*—are generally consumed [47].

Data on the collection site and Fx content of 24 major brown algae are presented in Table 1 [48,49,50,51,52,53,54,55,56,57,58,59,60,61,62,63,64,65]. The Fx profile of *U. pinnatifida* has been investigated by many researchers worldwide. Among these reports, seasonal variations in Fx content have been well observed in *U. pinnatifida* collected from Japan, New Zealand and Australia: Japan, 0.3–5.3 mg Fx/g dry weight (dw); New Zealand, 0.8–6.2 mg Fx/g dw; Australia, 2.1–3.0 mg Fx/g dw in juvenile and 1.3–2.9 mg Fx/g dw in adult sporophytes [48,50,51,52,53]. The highest Fx content in *U. pinnatifida* was observed in the winter in Japan, New Zealand and Australia. Furthermore, the Fx content of *S. horneri* peaked at 10.8 mg Fx/g dw in winter in Japan [50]. Moreover, although the collection period was unknown, the Fx level of *H. elongata* was surprisingly high at 18.6 mg Fx/g dw [62]. In general, the winter season, which is characterized by low levels of both sunshine duration and seawater temperature, facilitates increased Fx production in brown algae through the xanthophyll-cycle pathway, involving the formation of Fx by upstream carotenoids [66,67]. These results suggest that *U. pinnatifida*, *S. horneri* and *H. elongata* collected in the winter period may be particularly good sources of Fx for human consumption (Figure 1).

While Fx can be synthesized chemically, the harmless extraction of Fx from biological materials is extremely promising from the perspective of accessibility, economy, environmental load and safety for food additive, cosmetic and pharmaceutical applications. When obtaining Fx through natural extraction, not only brown algae but also microalgae are convenient for use in the large-scale preparation of various materials requiring Fx addition. For instance, the Fx levels of *Mallomonas* sp. SBV13, *Phaeodactylum tricornutum*, *Odontella aurita* and *Isochrisis* affinis *galbana*, all microalgae, are 26.6, 8.6–24.2, 21.7 and 18.2 mg/g dw, respectively [68,69,70]. Edible oil-, emulsion- or encapsulation-based Fx products in addition to pure Fx powder have been successfully commercialized worldwide. Their Fx-loading materials correspond to the protective shell are good options for achieving high bioavailability and health benefits of Fx in humans compared with a free body of Fx powder that get easily be degraded by light, temperature and oxidation. Edible oils, such as palm, olive and soybean oils, have low toxicity for humans and are often used to the extraction of Fx from brown algae or microalgae [71]. Long- and medium-chain triacylglycerols (TAGs), indigestible oils, Arabic gum and lecithin are frequently used as emulsifiers of Fx. Encapsulation has been constructed by the individual or combination with proteins, oligosaccharides, polysaccharides or glycolipids [72].

Many researchers have described the stability and functional properties of Fx-incorporated drinks, edible oils and foods [73,74,75,76,77]. Taken together, these findings suggest that the ingestion approaches of Fx for humans include not just plain brown algae but also Fx-incorporated products intended for human health benefits (Figure 1).

## 3. Effect by Brown Algae and Fx in CRC Cellular Lines

Many studies have shown an antiproliferative effect and the induction of apoptosis by brown algal extract, Fx and FxOH in CRC cells. It was suggested that ethanol extract of *U. pinnatifida* sporophyll induced apoptosis in human CRC HCT116 cells with the activation of caspase-3, unlike molecular mechanisms of cell death due to two anti-carcinostatic drugs (5-fluorouracil (5-FU) and irinotecan) [78]. Ethanol extract from the brown algae *Dictyopteris undulata* augmented endoplasmic reticulum stress, abrogated mitochondrial membrane potential and induced apoptosis in human CRC SW480 cells through the enhancement of Bax; active caspase-3, caspase-9 and caspase-12; phospho-PERK; phospho-IRE1; cleaved ATF6; CAAT/enhancer-binding protein-homologous protein; and attenuation of Bcl-2 [79,80]. Furthermore, methanol extract of the brown algae *Pylaiella littoralis* also had an apoptosis-inducing effect in HT-29 cells, with downregulation of Bcl-2, and upregulations of Bax, active caspase-3, cleaved PARP, phospho-JNK, phospho-ERK and p38 was observed [81]. Ethanol extracts of the two brown algae *T. ornata* and *P. pavonia* significantly suppressed the growth of HCT116 cells in a dose-dependent manner [82]. Mhadhebi et al. showed that the organic fraction from *Cystoseira sedoides* exerted an anti-proliferative effect in human CRC HCT15 cells in a dose-dependent manner (25–500 μg/mL), along with antioxidation and anti-inflammation effects [83]. Our group recently investigated the transcriptome profile and protein expression in human CRC DLD-1 cells with FxOH treatment. FxOH (5.0 μM) significantly reduced cell growth and induced apoptosis. Gene hierarchical clustering of the cells revealed a significant difference in 807 genes compared with control cells. The genes belonging to cancer-related pathways, including the cell cycle, integrin, PI3K/AKT, MAPK, nuclear factor erythroid 2 [NF-E2]-related factor 2 (NRF2), adipogenesis, TGF-β, signal transducer and activators of transcription (STAT) and wingless/integrated (WNT)/β-catenin signals, were remarkably altered. In addition, the protein expression of cyclin D1, cyclin D2, integrin α5, integrin β1, phospho-Paxillin(Tyr^31^), phospho-AKT(Ser^473^), phospho-C-Raf (Ser^338^), phospho-MEK1/2(Ser^217/221^), PPARγ and phospho-Smad2(Ser^465/467^) was downregulated, while that of phospho-ERK1/2(Thr^202^/Tyr^204^) and NRF2 was upregulated [84]. We also demonstrated the suppressive effect of CLIC4 signal and induction of anoikis due to attenuation of an integrin signal by FxOH in DLD-1 cells [85,86]. Tamura et al. showed that FxOH (10 μM) induced apoptosis in HCT116 cells by increasing the NF-κB activity. However, the co-treatment of FxOH and an NF-κB inhibitor enhanced apoptosis induction [87]. Lopes-Costa et al. reported that Fx reduced cell viability and induced DNA damage in HCT116 and HT-29 cells, while little apparent effect on normal human colon CCD-18Co cells was observed, except at high concentrations (50 and 100 μM). This report also showed that Fx enhanced the cytotoxic effect of 5-FU in HCT116 cells [88]. Fx treatment (50 and 75 μM) induced apoptosis in human CRC WiDr cells through the arrest of the G0/G1 phase of the cell cycle and upregulation of p21^WAF1/Cip1^, an inhibitor of cyclin D [89]. Both Fx and FxOH addition induced apoptosis in human CRC Caco-2 cells. The Bcl-2 protein expression was decreased on cells treated with Fx [90,91]. Fx suppressed cell growth in human CRC SW-620 s via the loss of adhesion and invasion activities and of MMP-9 expression and amplified the 5-FU-induced anti-proliferative effect [92]. Treatment with FxOH and Fx (both 20 μM) significantly inhibited and tended to inhibit cell growth, respectively, of primary cells isolated from tumor tissue from patients with CRC [93]. 

CCSCs are involved in the development of CRC and have properties that include self-renewal, pluripotency, chemotherapy resistance, sphere formation and tumorigenesis [94,95]. Sphere-forming cells prepared by using stem cell medium with slight growth factors from CRC cells, colonospheres (Csps), are known to possess CCSCs-like features [96]. Our previous study demonstrated that FxOH treatment significantly disintegrated Csps in a dose-dependent manner. In addition, FxOH downregulated phospho-AKT(Ser^473^), PPARβ/δ and PPARγ in the Csps and significantly suppressed subcutaneous tumorigenesis in NOD-SCID (NOD.CB17-*Prkd*c^scid^/J) mice [97]. FxOH also inhibited cell migration and invasion and induced apoptosis under both normoxia and hypoxia conditions by altering certain signals, including EMT, integrin, MAPK, WNT/β-catenin, apoptosis and/or STAT signals [98,99]. Similarly, Fx treatment exerted a sphere-forming activity in spheroid prepared from human breast cancer MCF-7 cells, although its molecular mechanisms remain unknown [100]. Further studies are needed to confirm the underlying mechanisms on the suppression of sphere formation in Csps by Fx and FxOH treatments. Table 2 summarizes the molecular mechanisms underlying the effects of brown algae and Fx in human CRC cells.

Clinical data on humans are the most important to support both the experimental findings and the hypothesized relationship between Fx and CRC. However, it is necessary to accumulate evidence on the anticancer effect of Fx for CRC in both in vivo and in vitro experiments as the basic mechanism for discussing the implications on potential effect of Fx in human.

## 4. Cancer Preventive Effect of Brown Algae and Fx in CRC Model Animals

### 4.1. Cancer Chemopreventive Effect of Whole Brown Algae and Fx-Containing Extract

Some researchers have reported on the anticancer properties of whole brown algae and Fx-containing extracts in CRC model animals. 

Recently, we cultivated *U. pinnatifida* on the Nesaki coast of Hokkaido, Japan, for 3 months and collected algae with the highest Fx content in February 2017 (Fx-high wakame, >5.0 mg Fx/g dw). Using a CRC murine model (azoxymethane–dextran sodium sulfate [AOM/DSS] mice with an ICR background), we investigated the effects of whole Fx-high wakame feeding on colorectal TME. The administration of Fx-high wakame (equivalent to Fx 30 mg/kg bw) for 7 weeks significantly decreased the number of CCSCs-like CD44^high^/EpCAM^high^ cells, CAFs-like αSMA^high^ cells, TAMs- and DCs-like CD206^high^ cells and increased apoptotic-cells-like cleaved caspase-3^high^ cells in colorectal mucosal tissue in the mice. In addition, the salivary glycine level was found to be a predictor correlated with the chemopreventive efficacy of Fx-high wakame in the mice [51]. Kong et al. showed that the administration of Fx-rich extract (1–5 g extract/kg bw) from *S. muticum* exerted antioxidation, anti-inflammation and/or anticancer effects in DSS-derived colitis-induced and AOM/DSS-induced BALB/c mice by reducing the disease activity score, nitric oxide (NO), malondialdehyde, TNF-α and IL-6 and increasing the level of SOD. In addition, the extract prevented the onset of colorectal tumors and restored lymphocyte proliferation and survival rates [101]. Son et al. revealed that diet feeding both 2% and 6% whole *H. fusiforme* for 8 weeks significantly reduced the number of aberrant crypt foci (ACF) and the rate of proliferating cell nuclear antigen labeling index in mucosal tissue of an AOM-induced F344 rat CRC model [102]. Mahmoud et al. investigated the CRC chemopreventive effect using ethanol extracts of two brown algae, *T. ornata* and *Padina pavonia*, collected from the shore of the Red Sea in Egypt. The administration of 100 mg/kg bw of either *T. ornata* or *P. pavonia* extract for 8 weeks in colorectal tissue of AOM-induced albino Swiss CRC mice significantly prevented colorectal inflammation and oxidation by reducing the lipid peroxidation and NO levels and downregulating the NF-κB expression and upregulating the peroxisome proliferator-activated receptor gamma (PPARγ) and p53 expression. In addition, the colorectal levels of GSH, SOD and GPx activities in the mice were significantly increased by the feeding of both *T. ornata* and *P. pavonia* extracts [82]. 

Of note, Das et al. prepared ethanol extract containing 15 mg Fx/g from *S. japonica* and administered it as an ad libitum dimethyl sulfoxide solution (final Fx concentration: 0.075 mg/mL) to AOM-initiated ddY mice for 4 weeks to investigate the difference in CRC chemoprevention from other groups given 0.05 and 0.1 mg Fx/mL solutions. Consequently, the administrations of *S. japonica* extract, 0.05 and 0.1 mg Fx/mL solutions all significantly decreased the number of colorectal ACF in the mice to similar degrees. These findings suggested that *S. japonica* extract exerted an inhibitory effect on the onset of colorectal ACF with the same potency as Fx alone [103]. Further complicating matters, Reddy et al. showed that the dietary intake of *Laminaria angustata* (Mitsuishikombu) (diet containing 10% algae) for 28 weeks significantly increased the incidence and multiplicity of colorectal tumors in an AOM-induced F344 rat CRC model [104].

### 4.2. Cancer Chemopreventive Effect of Fucoxanthin Itself

Some researchers have studied the direct anticancer property of Fx in CRC model animals. Our group previously found a cell death mechanism by which FxOH treatment induced anoikis in human CRC cells [86]. Anoikis is an apoptotic mechanism that occurs following cell detachment due to a lack of integrin anchor points between the cell and the extracellular matrix (ECM) or the cell and neighbor cells, and it is physiologically significant for tissue homeostasis and disease. During the anoikis process, certain core mechanisms, such as phosphatidylinositol-3 kinase/protein kinase B (PI3K/AKT), MAPK and transforming growth factor-beta (TGF-β) signals, are first inhibited by changes in some transmembrane receptors, and cells subsequently become detached from the ECM, finally inducing caspase activation, which triggers anoikis. Cancer cells frequently suppress this stepwise anoikis induction. Therefore, anoikis resistance in cancer cells is indispensable for the cell survival, epithelial–mesenchymal transition (EMT), invasion and metastasis [105,106,107]. 

Following our anoikis findings in vitro, we explored whether or not Fx administration in an AOM/DSS-induced ICR mouse model of CRC induced anoikis in the colorectal tissue. As a result, oral Fx administration (30 mg/kg bw) for 2 weeks before sacrifice significantly suppressed the number and size of colorectal tumors by enhancing anoikis-like integrin β1^low/-^, phospho(p)-FAK(Tyr^397^)^low/-^ and pPaxillin(Tyr^31^)^low/-^ with cleaved caspase-3^high^ cells in the colorectal mucosal crypts [108]. Furthermore, Fx treatment also markedly increased the number of anoikis-like integrin β1^low/-^/cleaved caspase-3^high^ cells in colorectal adenocarcinoma in the mice [109]. These two reports suggest that the induction of anoikis by Fx was the relevant anticancer mechanism in AOM/DSS mice. CCSCs often promote an EMT accompanied by the alterations of related proteins. EMT is considered to mediate their migration and invasion of the cells and to cause CRC recurrence and distant metastasis [110]. Therefore, the inhibition of the EMT phenotype in CCSCs would be a promising approach for cancer prevention. We speculate that the anoikis induction in colorectal normal mucosa and adenocarcinoma of AOM/DSS mice with Fx treatment lead to suppress the occurrence and EMT activation of CCSCs.

The formation of TME is a significant process in the onset of adenocarcinoma in colorectal mucosal tissue [20,21,22]. We previously showed that Fx administration (30 mg/kg bw) for 8 and 14 weeks significantly prevented colorectal carcinogenesis and decreased the number of CCSCs-like CD44^high^/EpCAM^high^ cells, CAFs-like αSMA^high^ cells, TAMs- and DCs-like CD206^high^ cells and/or increased apoptotic cell-like cleaved caspase-3^high^ cells in colorectal mucosal tissue in AOM/DSS mice. In addition, the salivary glycine level was found to be a predictor of the chemopreventive efficacy of Fx in the mice [111,112]. These findings suggested that Fx was a promising inducer for the occurrence suppression in CCSCs of the mice.

Notably, changes in the gut microbiota can also function as a key trigger in human colorectal carcinogenesis [15,113,114]. We investigated the chemopreventive potency of Fx and its effect on the gut microbiota in AOM/DSS mice. Oral Fx administration (30 mg/kg bw) for 14 weeks significantly prevented the onset of colorectal adenocarcinoma in mice with attenuations of Bacteroidlales and Rikenellaceae and enhancement of Lachnospiraceae in the fecal microbiota composition. Of note, the oral administration of a fecal suspension from the Fx-treated mouse suppressed the number of colorectal adenocarcinoma in another AOM/DSS mice with a successful increase of gut Lachnospiraceae. These findings suggested that the induction of changes in the gut microbiota by Fx is a significant mechanism underlying the cancer chemopreventive effects of Fx in AOMDSS mice [115].

CCSCs occupy only a small subset of CRC tissue, but they are thought to play an important role in cancer progression. Self-renewal, sphere formation, differentiation, and tumorigenicity in animals have been characterized as biological properties for CCSCs [94,95]. Therefore, we especially focused on the effects of Fx and FxOH on the tumorigenesis from CCSCs in two immunodeficiency murine models. Consequently, we have revealed that the administration of Fx and FxOH inhibited tumorigenesis in xenograft NOD/SCID and BALB/c mice, respectively, subcutaneously injected with CCSCs-like cells prepared from human CRC HT-29 cells. In addition, the administration of Fx to BALB/c mice significantly downregulated the cyclin D1 expression in the tumor [97,116]. Furthermore, we investigated the effect of Fx in a *Apc*^Min/+^ mouse model of human FAP. The intake of Fx diet (1000 ppm) for 5 weeks significantly attenuated the number of colorectal adenocarcinoma lesions in DSS-treated *Apc*^Min/+^ mice with downregulation of cyclin D1 expression in mucosal tissues [117]. One group described the effects of Fx in another animal model: Kim et al. showed that Fx administration in drinking water (0.01%) for 7 weeks significantly suppressed the onset of colorectal ACF and the BrdU Labeling Index in the colorectal crypt compartment in a 1,2-dimethylhydrazine-induced murine CRC model (B6C3F_1_) [41]. 

Many reports have described the cancer chemopreventive effects seen in animal CRC models treated with whole brown algae, extract containing Fx and Fx itself. However, the detailed molecular mechanisms underlying these effects in the colorectal tissue of animal models remain elusive. Further investigations are needed to determine the CRC chemopreventive effects of brown algae and Fx.

### 4.3. Effect by Brown Algae and Fx in CRC Risks

**Figure 2:**
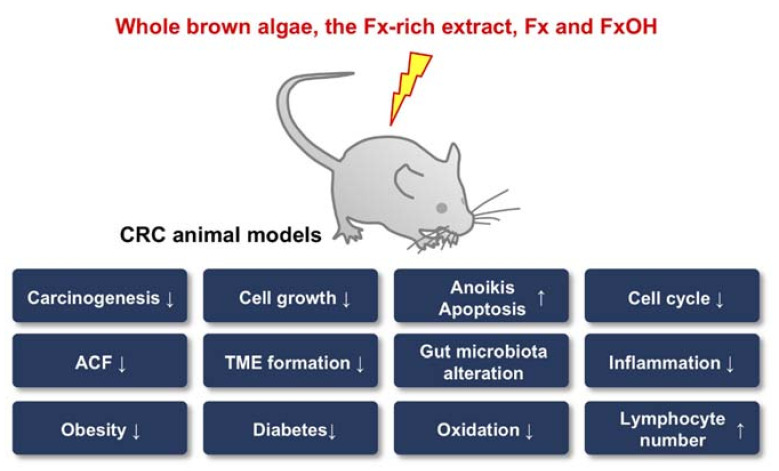
Possible mechanisms underlying the cancer chemopreventive effects of whole brown algae, fucoxanthin (Fx)-rich extract, Fx and fucoxanthinol (FxOH) against colorectal cancer (CRC). ACF, aberrant crypt foci; TME, tumor microenvironment. ↑, induction or increase; ↓, inhibition or decrease.

Many researchers have reported the attenuation of CRC risk factors, such as anti-obesity, anti-metabolic syndrome and anti-inflammation effects, and changes in gut microbiota by whole brown algae, Fx-containing extracts and Fx itself in animal models. Diets containing *U. pinnatifida* ethanol extracts, equivalent to 0.05% and 0.2% Fx, which has already demonstrated anti-obesity effects when administered through the diet in C57BL/6N mice, or another diet containing *S. horneri* powders (2% and 6%) reduced both the body and WAT weights in C57BL/6J mice fed a high-fat diet, with enhancement of the UCP-1 mRNA expression in WAT [118,119,120]. Okada et al. also reported on the anti-obesity effects, characterized by reductions in the body and WAT weights, changes in serum lipid profiles and upregulation of the UCP-1 protein and mRNA expression in KK-A^y^ mice with *U. pinnatifida* lipid treatment (0.2% as a drink; 1.0% as a diet) [73]. Grasa-López et al. revealed the anti-obesity and anti-inflammation effects of both *U. pinnatifida* (400 mg/kg bw) and Fx (1 mg/kg bw) in Wister rats given a high-fat diet, along with the upregulation of PPARα, PPARγ coactivator-1α, PPARγ and UCP-1 and downregulation of IL-6 [34]. Diet containing *S. polycystum* (2.5–10.0%) induced significant decreases in the BW and plasma LDL-cholesterol and TAG levels and increased the high-density lipoprotein-cholesterol levels in Sprague-Dawley rats fed a high-fat diet [121]. The oral administration of extracts from two brown algae—*P. pavonia* and *T. ornata* (100 mg/kg bw)—for 21 d exerted antioxidation and anti-inflammation effects in streptozotocin/nicotinamide-induced type 2 diabetic rats (albino) through the reductions in glucose, TNF-α and malondialdehyde, and increases of insulin, GSH, GPx and SOD [122]. An ethanol extract of the brown algae *Petalonia binhamiae* (150 mg/kg bw) for 70 d induced an anti-obesity effect with many obesity-related markers in high-fat-diet-fed C57BL/6 mice [123]. Maeda et al. showed that the administration of diets containing Fx-rich *U. pinnatifida* extract (2.0%) significantly reduced the WAT weight in both Wister rats and KK-A^y^ mice, but not the BAT weight. In addition, both a 2.0% *U. pinnatifida*-containing diet and 0.4% Fx diet induced the marked upregulation of the UCP1 expression in WAT of KK-A^y^ mice compared with that in control mice [124]. Similarly, those authors further demonstrated the anti-obesity and anti-diabetic effects of Fx in KK-A^y^ and C57BL/6J mice [125,126,127,128,129,130]. Other researchers have described the anti-diabetic effect of Fx and *U. pinnatifida* extract in C57BL/6N and C57BL/6J mice, respectively [131,132]. Furthermore, treatment with Fx (50 and 100 mg/kg) could exert the anti-inflammatory effect with BW reduction and colorectal mucosal damage, decreases of PGE2, COX-2 and NF-κB in a DSS-induced colitis murine model [133]. Another group reported on changes in the gut microbiota in the administration of whole brown algae. The intake of a diet containing 10% dried *U. pinnatifida* or *S. japonica* for 4 weeks increased the *Prevotella*, *Alistipes* and Bacteroides genera and decreased the *Roseburia*, *Mollicute* and *Oscillibacter* genera in feces of Sprague-Dawley rats [134]. Another two groups explored the alteration of the gut microbiota in obese mice by Fx. Sun et al. showed that the administration of a high-fat diet containing Fx (1 mg Fx/g diet) decreased the rate of Lachnospiraceae and Erysipelotrichaceae and increased the rates of *Lactobacillus*, *Lactococcus*, *Bifidobacterium* and several butyrate-producing bacteria in feces of an obese murine C57BL/6J model, along with decreasing serum levels of TNF-α and IL-6. In addition, the gut bacterial taxa were significantly associated with obesity phenotypes and the degree of inflammation [135]. Another group reported that Fx administration (125 mg/kg bw) for 4 weeks reduced BW and changed the cecal and fecal microbiota in BALB/c mice given a normal or a high-fat diet. In particular, changes in the ratio of Firmicutes/Bacteroidetes and the composition of *S24-7* and *Akkermansia* were observed in the cecal contents [136]. Furthermore, Liu et al. prepared a fecal bacterial suspension from C57BL/6 mice served a normal diet. The bacteria were cultured under anaerobic conditions and treated with 0.1 mg/mL Fx. The Fx treatment consequently suppressed the growth of *Escherichia coli* but promoted that of *Lactobacilli* [137]. Figure 2 summarizes the molecular mechanisms underlying the effects of whole brown algae, Fx-rich extract and Fx on CRC itself or its risk factors in animal models.

## 5. Beneficial Effects of Brown Algae and Fx in Human

### 5.1. Effects of Fx and Fx-Containing Materials on the Risk Factors Associated with CRC

Little information is available on the direct mechanisms underlying the anti-CRC properties of Fx and Fx-containing materials. However, interventional studies have shown that whole brown algae, Fx extracts and Fx itself can improve the risk factors associated with CRC, such as obesity, diabetes, metabolic syndrome, inflammation and oxidation. 

Obesity is a chronic metabolic disorder defined as excessive visceral fat accumulation, typically characterized by a body mass index (BMI) of ≥ 30 kg/m^2^ (≥25 kg/m^2^ in Japan), an increased waist circumference and/or a reduced energy expenditure [138]. Large cohort studies have described a positive association between obesity and CRC [139,140] and between diabetes and CRC [141,142]. Obesity is mainly caused by overeating, low physical activity and heritable features. The disease leads to low-level inflammation and metabolic syndrome, such as hyperlipidemia, hypertension, diabetes mellitus, non-alcoholic fatty liver disease (NAFLD) and arteriosclerosis. The excessive fat accumulation is accompanied by invasion of inflammatory cells into adipose tissue, which secretes cancer-related adipokines/chemokines including adiponectin, plasminogen activator inhibitor-1 (PAI-1), leptin, resistin, tumor necrosis factor-α (TNF-α), monocyte chemoattractant protein-1 (MCP-1), nicotinamide phosphoribosyltransferase (NAMPT), interleukine-6 (IL-6) and lipocalin 2 [143,144,145,146,147]. 

Inflammation is a protective response that causes injured tissues to heal. However, chronic inflammation induced by the persistent activation of signaling pathways without normal healing is a major CRC risk factor [148,149,150]. The core inflammatory mediators in white blood cells and tissue cells, such as nuclear factor-κB (NF-κB), cyclooxygenase-2 (COX-2), prostaglandin E2 (PGE2), peroxisome proliferator-activated receptor δ (PPARδ), IL-6, mitogen-activated protein kinase (MAPK), c-Jun NH_2_-terminal kinases and activating protein-1, lead to carcinogenesis; therefore, the suppression of these inflammatory activators is a significant target for anti-inflammatory agents [151,152,153]. 

Oxidative stress resulting from increased production of reactive oxygen species (ROS) by various stimuli is also strongly associated with the development of CRC and IBD [154,155,156,157,158,159,160]. Enhanced oxidative stress induces increased DNA base oxidation (8OHdG-8hydroxy-2’-deoxyguanosine) and lipid peroxidation under conditions of inflammation and carcinogenesis in human subjects [161,162]. The ROS superoxide (O_2_^-^), hydroxyl radicals (HO^・^) and hydrogen peroxide (H_2_O_2_) are mainly generated through the mitochondrial electron transport chain via enzymatic and non-enzymatic pathways, and their byproducts are balanced by glutathione (GSH) and antioxidant enzymes, such as superoxide dismutase (SOD), catalase, glutathione peroxidase (GPx), peroxiredoxin or antioxidant agents, to achieve a normal cell function [163]. However, excessive ROS generation frequently plays a major role in tumorigenesis [164,165]. 

As an early experiment, Adidov et al. investigated the anti-obesity effect of the Fx-containing oil Xanthigen^TM^ (PL Thomas & Co., Morristown, NJ, USA) in humans. The consumption of Xanthigen-600, which is composed of an algal extract (equivalent to 2.4 mg Fx) and plant oil, by obese premenopausal women with NAFLD or normal liver fat content for 16 weeks significantly decreased their BW, waist circumference, body and liver fat content, liver enzymes, serum TAG and/or C-reactive protein levels [166]. Mikami et al. prepared 1 and 2 mg Fx capsules composed of an algal oil extracted from *S. horneri*, medium-chain triacylglycerol, lecithin and vitamin E. The administration of 2 mg Fx/d for 8 weeks significantly decreased the HbA1c level compared with the placebo control. They investigated the polymorphism status of uncoupling protein 1 (UCP1) in the subjects [33]. UCP1, a mitochondrial membrane protein, plays a central role in the metabolic thermogenesis process for inhibiting excessive fat accumulation [167]. The UCP1 molecule is strongly expressed in brown adipose tissue (BAT), which promotes whole body energy expenditure, and its protein aberration leads to the development of obesity, although most fat is accumulated in the white adipose tissue (WAT), where UCP1 is absent. Of note, UCP-1 can be induced by various intrinsic and extrinsic stimuli [168,169,170]. Maeda et al. demonstrated that the intake of a diet containing Fx-rich *U. pinnatifida* extract significantly reduced the WAT weight with the enhancement of UCP1 expression in KK-A^y^ mice [124]. Interestingly, the reduction of HbA1c in subjects with the UCP1-3826 G/G genotype was significantly greater than that in those with an A/A or A/G genotype. No significant change in the HbA1c level was observed following 1 mg Fx administration [33]. A capsule of phlorotannin (107.3 mg) and Fx (36.5 mg)-rich content was prepared from ethanol extract of *A. nodosum* collected on the coast of the United Kingdom and administered to individuals with a BMI ≥ 25 for 8 weeks. As a result, the algal extract slightly reduced the DNA damage in these obese individuals [171]. 

However, some researchers have described the beneficial effects for humans of whole brown algae or its extract with unknown Fx contents. The intake of 0.9 g *A. nodosum* for 6 weeks reduced the BW, BMI and TAG level and enhanced the adiponectin levels in healthy volunteers [172]. A single dose of both *U. pinnatifida* itself (4 g) and its sporophylls (70 g) significantly decreased the postprandial glucose concentration in plasma of healthy volunteers at 0.5 h after a meal [173,174]. Dosage plan of 4 g/d for 4 weeks plus 6 g/d for 4 weeks of *U. pinnatifida* powder-packed capsules brought about reductions in the systolic blood pressure and waist circumference in participants with at least one symptom of metabolic syndrome [175]. A daily intake of 50 g of a snack food containing 64 mg of whole *U. pinnatifida* for 8 weeks significantly reduced the total cholesterol, low density lipoprotein (LDL)-cholesterol and resistin levels [176]. The administration of fermented *S. japonica* (1.5 g/d) for 4 weeks significantly downregulated the activities of catalase and SOD [177]. The intake of *S. japonica* (6 g/d for 4 weeks) also improved the molecular species profiles of phosphatidylcholine (PC), phosphatidylethanolamine (PE), lysophosphatidylcholine and lysophosphatidylethanolamine as well as the free fatty acid levels, and algal ingestion increased the plasmanyl and plasmenyl forms of PC and PE in the serum of participants with abnormal serum triacylglycerol levels [178,179]. Furthermore, dietary intervention of *S. japonica* (2 g/d for 6 weeks) reduced the body fat proportion and enhanced the adiponectin level in healthy volunteers [180].

Brown algae contain many attractive hydrophilic and lipophilic compounds with nutritional benefits for both humans and animals. The promising agents considered to be involved in these effects include Fx, fucosterol, polyunsaturated fatty acids, polysaccharides, peptides, bromophenols, phlorotannins and assorted minerals [181,182,183]. Therefore, it must be noted that the beneficial effects of whole brown algae and its extract in human subjects are not due to Fx alone. 

### 5.2. Effects of Fucoxanthin Itself on CRC Risk

Abidov et al. have demonstrated that administration of 2.4–8.0 mg Fx for 16 weeks enhanced the resting energy expenditure in obese volunteers with NAFLD but had no effect at 1.6 mg [166]. Hitoe et al. investigated the anti-obesity effect of the commercial Fx oil Fucoxanthin-P1 containing a powder with 1% Fx (Oryza Oil & Fat Chemical Co. Ltd., Osaka, Japan) in Japanese adults with a BMI ≥ 25 kg/m^2^. Consequently, the interventional intake of 3 mg Fx/d for 4 weeks significantly reduced the BMI, fat areas (total, subcutaneous and visceral parts) and waist circumference compared with placebo groups. In addition, the administration of 1 mg Fx/d for 4 weeks decreased the total fat area alone [32].

In summary, interventional studies, a prospective cohort study and two case-control studies on the association between the intake of seaweed likely containing brown algae and CRC have shown promising findings concerning the effect of brown algae intake on preventing CRC. In addition, the daily intake of >0.9 g of whole brown algae or 2 mg of Fx may result in anti-obesity, anti-metabolic syndrome, antioxidation and anti-inflammation effects. To date, there is little information available concerning interventional studies of brown algae and Fx for gut microbiota and hereditary CRC syndromes. Further work is needed to confirm the response in patients with CRC or CRC risks. 

The interventional results concerning whole brown algae, Fx-rich extracts and purified Fx are summarized in Table 3.

### 5.3. Clinical Studies with Whole Brown Algae and Fx-Containing Extracts in CRC

Hoshiyama et al. showed that a high intake of seaweed, likely including *U. pinnatifida*, showed a significant trend toward an inverse association with the risk of overall CRC (odds ratio (OR), 95% confidence interval (CI) = 0.2 [0.0–0.9], P_trend_ < 0.01) and rectal cancer (OR, 95% CI = 0.4 [0.1–1.1], P_trend_ = 0.01) as a case-control study in Japan [184]. In addition, Minami et al. investigated the association between the Japanese food intake and major digestive tract cancers from 1997 to 2013 as a prospective cohort study at an institution in Japan. As a result, the intake of seaweed, likely including *U. pinnatifida*, tended to be inversely associated with death in patients with rectal cancer (P_trend_ = 0.02) [185]. Kim et al. reported a significant inverse association between dietary intake of algae containing *U. pinnatifida* and CRC in a case-control study of Korean patients (OR, 95% CI = 0.65 [0.50–0.85]). In addition, the rs6983267 polymorphism of c-MYC, an oncogene, was associated with a significant interaction between the dietary algae intake and both the overall CRC and rectal cancer risk [186]. Epidemiological data in humans supporting the role of Fx in CRC prevention are few and not well-addressed. They involve the same geographic area, Japan [184,185] and Korea [186]. Although there are no or few specific algae among those listed in Table 1, they contain Fx at different amounts. Indeed, the sample size is relatively small, but epidemiological studies [185,186] may suggest that dietary seaweed containing Fx have a positive benefit as a chemoprevention and/or chemotherapeutic agent for CRC risk.

## 6. Conclusions

The high-polarity xanthophyll Fx is specific to brown algae, and microalgae and has been verified as safe without side effects in human [24,32,166]. This review discussed the most recent studies available concerning Fx as a potentially useful agent for CRC prevention. 

Edible brown algae are often high in Fx content, and their consumption has been expanding in Asia, North and South America and Europe. In particular, the three algae, *U. pinnatifida*, *S. horneri* and *H. elongata*, are not only commonly eaten all around the world, but they have also been proven considerable Fx sources (Table 1). Brown algae are characterized by increased Fx production under low-sunlight conditions with cold seawater temperatures. Therefore, *U. pinnatifida*, *S. horneri* and *H. elongata* collected during the period of high Fx production may be useful as potent sources of Fx. In addition, the acquisition of Fx from not only brown algae but also microalgae can be convenient on large-scale preparation for food, cosmetic and pharmaceutical applications (Figure 1). 

Fx-rich extract, Fx and FxOH can induce apoptosis and anoikis in human CRC cells and their spheroids through a number of molecular mechanisms. Such treatments can alter the activities and protein expression of caspases, Bax, Bcl-2, PARP, JNK, MAPK, PI3K/AKT, integrin, WNT/β-catenin, STAT and PPARs and induce changes in the cell cycle, DNA damage and the CRC cellular functions of adhesion, migration and invasion, as well as the mitochondria and endoplasmic reticulum of these cells (Table 2). A few molecular alterations in human CRC cells were correspondingly observed in CRC animal models treated with whole brown algae, its Fx-rich extract or Fx itself. Consecutive studies in vitro will be important as the basis for clarifying the molecular mechanisms underlying cancer prevention in humans with CRC and CRC animal models by brown algae and Fx. Further studies are needed to confirm the anticancer mechanisms in both CRC animal models and cells. 

CRC animal models have been the vehicle for many discoveries concerning the anticancer effects of whole brown algae, its Fx-rich extract and Fx itself. The TME, inflammation, oxidation and gut microbiota, which are significant factors associated with colorectal carcinogenesis, have been reported to be prime targets of Fx and were found to be altered by this agent’s cancer chemopreventive properties. In addition, the administration of Fx induced anoikis in CRC animal models (Figure 2). However, the detailed molecular mechanisms underlying the cancer chemopreventive effect in animals remain poorly understood. 

Few reports on the direct anticancer effects of Fx intake on human CRC have been published. However, the negative association between the intake of seaweed likely including brown algae and human CRC has been reported. IBD, heritable factors, obesity, diabetes, metabolic syndrome, inflammation and oxidation are suggested as key risks triggering colorectal carcinogenesis. Interventional approaches have revealed that the administration of whole brown algae, its Fx-rich extract and Fx itself can ameliorate most of these CRC risks (Table 3). However, the underlying mechanisms remain elusive. Further clinical investigations are needed to assess the anticancer effect of Fx in humans. 

Finally, this review suggests that whole brown algae, its Fx-rich extract, Fx and FxOH may be potential candidates as beneficial agents for preventing CRC.

## Figures and Tables

**Table 1:** Fucoxanthin content in 24 major edible brown algae worldwide.

Family	Species	Common Name	Synonym Name	Collected Location	Body Part of Alga	Fx (mg/g dw) ^a^	Reference
Alariaceae	*Undaria pinnatifida*	Wakame		Japan New Zealand Australia	Blade Blade --- ^b^	0.3–5.3 0.8–6.2 1.3–3.0	[48,50,51] [52] [53]
	*Alaria esculenta*	Dabberlocks		Ireland	Blade	0.9	[54]
	*Alaria crassifolia*	Chigaiso		Japan	Blade	1.1	[49]
Sargassaceae	*Sargassum horneri*	Akamoku		Japan Korea	Lateral branch ---	0.8–10.8 0.8	[49,50] [55]
	*Sargassum fusiforme*	Hiziki		Japan	Lateral branch	1.1	[49]
	*Sargassum wightii*			India	---	0.1	[56]
	*Sargassum binderi*			India	---	0.7	[57]
	*Sargassum duplicatum*			India	---	1.0	[57]
	*Nizamuddinia zanardinii*			Iran	---	0.6–1.7	[58]
	*Cystoseira indica*			Iran	---	2.3–3.6	[58]
	*Turbinaria ornata*			Indonesia	---	1.3	[59]
Laminariaceae	*Saccharina japonica*	Makombu	*Laminalia japonica*	Japan China Korea	--- --- ---	0.2 0.4 0.5	[60] [61] [55]
	*Saccharina sculpera*	Gagomekombu	*Kjellmaniella crassifolia*, *Saccharina crassifolia*	Japan	Lateral branch	0.7	[49]
	*Laminaria digitata*			Ireland	Blade	0.7	[54]
	*Laminaria saccharina*	Sugar kelp, sea belt	*Fucus saccharinus, Saccharina latissima*	Ireland	Blade	0.5	[54]
Lessoniaceae	*Ecklonia kurome*	Kurome		Japan	Blade	1.7	[59]
Fucaceae	*Fucus vesiculosus*	sea oak		Ireland	Blade	0.7	[54]
	*Fucus serratus*	Toothed wrack		Ireland	Blade	0.3	[54]
	*Ascophyllum nodosum*	Rockweed		Ireland	Blade	0.4	[54]
Ralfsiaceae	*Analipus japonicus*	Matsumo		Japan	Lateral branch	1.4	[49]
Chordariaceae	*Sphaerotrichia divaricata*	Kusamozuku		Japan	Whole body	0.2	[49]
Himanthaliaceae	*Himanthalia elongata*	Sea spaghetti	*Fucus elongatus, Himanthalia lorea*	Ireland Spain	Blade	0.3–18.6 1.1	[54,62] [63]
Chordariaceae	*Cladosiphon okamuranus*	Okinawamozuku		Japan	---	0.3	[64]
Dictyotaceae	*Padina australis*			Malaysia Indonesia	--- ---	0.4 1.3	[65] [59]

^a^ Variations of fucoxanthin (Fx) content in major edible brown algae include seasonal change and different location {value: minimum–max mg/g dry weight (dw)}. ^b^ The body part for extraction in the alga is unknown.

**Table 2:** Effect of fucoxanthin (Fx)-rich brown algal extract, Fx and fucoxanthinol (FxOH) in human colorectal cancer cell lines.

Brown Algal Extract or Compound (Additive Concentration)	Cell Line (Cell Type)	Promoted Molecular Mechanism(s)	Involved Intracellular Component	Final Outcome (Cell Function)	Reference
Ethanol extract of *Undaria pinnatifida* sporophyll (~2.0%)	HCT116 (PCs)	Caspase-3 activation, and non-oxidative mechanisms differed from those of 5-fluorouracil and irinotecan treatments	NA	Apoptosis	[78]
Ethanol extract of *Dictyopteris undulata* sporophyll (~200 μg/mL)	SW480 (PCs)	Augmentation of endoplasmic reticulum stress; attenuation of mitochondrial membrane potential; increases of Bax, caspase-3, caspase-9, caspase-12, phospho-PERK, phospho-IRE1, cleaved ATF6, and CAAT/enhancer-binding protein-homologous protein; and decrease of Bcl-2	Endoplasmic reticulum, and mitochondria	Apoptosis	[79,80]
Methanol extract of *Pylaiella littoralis* (~100 μg/mL)	HT-29 (PCs)	Attenuation of mitochondrial membrane potential, decrease of Bcl-2, and increases of Bax, active caspase-3 form, cleaved PARP, phospho-JNK, phospho-ERK and p38	Mitochondria	Apoptosis	[81]
Ethanol extracts of *Turbinaria ornata and Padina pavonia* (~50 μg/mL)	HCT116 (PCs)		NA	Growth inhibition	[82]
Organic fraction of *Cystoseira sedoides* (~500 μg/mL)	HCT115 (PCs)		NA	Growth inhibition, antioxidation and anti-inflammation	[83]
FxOH (~5.0 μM)	DLD-1 (PCs)	Alterations of gene set belonging cell cycle, integrin, PI3K/AKT, MAPK, NRF2, adipogenesis, TGF-β, STAT and WNT/β-catenin signals, decreases of cyclin D1, cyclin D2, integrin α5, integrin β1, phospho-Paxillin(Tyr^31^), phospho-AKT(Ser^473^), phospho-C-Raf (Ser^338^), phospho-MEK1/2(Ser^217/221^), PPARγ and phospho-Smad2(Ser^465/467^), and increases of phospho-ERK1/2(Thr^202^/Tyr^204^) and NRF2	NA	Apoptosis	[84]
FxOH (~5.0 μM)	DLD-1 (PCs)	Arrest of G2/M cell cycle phase, decreases of CLIC4, integrin β1, phospho-Smad2(Ser^465/467^) and NHERF2	NA	Apoptosis	[85]
FxOH (~2.5 μM)	DLD-1 (PCs)	Alteration on cellular distribution of integrin β1, and decreases of phospho-FAK(Tyr^397^), phospho-AKT(Ser^473^) and PPARγ	NA	Anoikis	[86]
FxOH (~10 μM)	HCT116 (PCs)	Arrest of G0/G1 cell cycle phase, activations of NF-κB and caspase-3, and increases of XIAP and cIAP-1	NA	Apoptosis	[87]
Fx (~100 μM)	HCT116 and HT-29 (Both PCs)	Increase of p53 and decrease of Bcl-2 in HCT116 cells, and increase of Bax and decrease of pro-caspase-9 in HT-29 cells	NA	Growth inhibition	[88]
Fx (~75 μM)	WiDr	Arrest of G0/G1 cell cycle phase, and increases of p21^WAF1/Cip1^ and p27^Kip1^, and decreases of phospho-pRb(Ser^780^), phospho-pRb(Ser^807/811^), cyclin D1, cyclin D2 and cyclin D3	NA	Apoptosis	[89]
Fx (~15.2 μM)	Caco-2	Decrease of Bcl-2 and activation of caspases	NA	Apoptosis	[90]
FxOH (~25 μM)	Caco-2		NA	Growth inhibition	[91]
Fx (~30 μM)	SW-620	Loss of adhesion and invasion activities, and decrease of MMP-9	NA	Growth inhibition	[92]
Fx and FxOH (~20 μM)	Primary cells in CRC patients		NA	Growth inhibition	[93]
FxOH (~5.0 μM in vitro, 5 mg/kg body weight in vivo)	HT-29 (Csps)	Decreases of phospho-AKT(Ser^473^), PPARβ/δ and PPARγ, suppression of tumorigenesis in NOD/SCID mice	NA	Apoptosis	[97]
FxOH (~50 μM)	HT-29 (Csps)	Suppressions of cell migration and invasion; attenuations of EMT, integrin, MAPK and STAT signal proteins; decrease of p53; and increase of active caspase-3 form	NA	Apoptosis under normoxia condition	[98]
FxOH (~50 μM)	HT-29 (Csps)	Attenuations of EMT, integrin, MAPK and STAT signal proteins; decreases of HIF-1α, cyclin D1and p53; and increases of phospho-β-catenin(Ser^31/37^/Thr^42^) and active caspase-3 form	NA	Apoptosis under hypoxia condition	[99]

Parent cells (PCs) indicate each intact cell line, most of which are the adherent types. Colonospheres (Csps) indicate a spheroid prepared from the parent cells by stem cell medium and some growth factors. Fx, fucoxanthin; FxOH, fucoxanthinol; NA, not available; PERK, protein kinase RNA (PKR)-like ER kinase; IRE1, inositol-requiring enzyme 1; ATF6, activating transcription factor 6; PARP, poly(ADP-ribose) polymerase; JNK, c-Jun NH2-terminal kinase; ERK, mitogen-activated protein kinase 1; PI3K/AKT, phosphatidylino-sitol-3 kinase/protein kinase B; MAPK, mitogen-activated protein kinase; NRF2, nuclear factor erythroid 2 [NF-E2]-related factor 2; TGF-β, transforming growth factor beta; STAT, signal transducers and activators of transcription; WNT, wingless/integrated; MEK, mitogen-activated protein/extracellular signal-regulated kinase; PPARγ, peroxisome proliferator activated receptor gamma; CLIC4, chloride intracellular channel 4; NHERF2, Na^+^/H^+^ exchanger regulatory factor 2; FAK, focal adhesion kinase; NF-κB, nuclear factor-κB; XIAP, X-linked inhibitor of apoptosis protein; cIAP-1, cellular inhibitor of apoptosis protein-1; MMP-9, matrix metallopeptidase 9; PPARβ/δ, peroxisome proliferator activated receptor beta/delta; NOD/SCID, NOD.CB17-*Prkd*c^scid^/J; EMT, epithelial-mesenchymal transition; HIF-1α, hypoxia-inducible factor-1 alpha.

**Table 3:** Effect of brown algae and fucoxanthin on CRC risk factors in human interventional studies.

Brown Algae Source	Fx Dosage	Administration Type	Study Design	Effect	Reference
Unknown	2.4 mg/d	A capsule of algal lipid-rich extract containing 300 mg pomegranate seed oil and 300 mg dw brown algal extract (Xanthigen^TM^)	Double-blind, placebo-controlled, randomized trial in 151 women with non-diabetic and obese premenopausal (period, 16 weeks)	Reductions of BW, waist circumference, body and liver fat contents, liver enzymes, serum TAG and C-reactive protein	[166]
*Sargassum horneri*	2.0 mg/d	A capsule containing 220 mg dw *S. horneri* Fx-rich extract	Single-blind, placebo-controlled, randomized trial in 60 adults (30–77 y) with normal-weight and obese (period, 8 weeks)	Reductions of blood HbA1c level	[33]
*Ascophyllum nodosum*	36.5 mg/d	A capsule containing 100 mg dw *A. nodosum* ethanol/water extract	Double-blind, placebo-controlled, randomized trial in 80 women (30–65 y) with obese (period, 8 weeks)	Weak inhibition of DNA damage	[171]
*Ascophyllum nodosum*	Unknown/d	A capsule containing 900 mg dw of whole *A. nodosum*	Double-blind, placebo-controlled, randomized trial in 43 healthy adults (21–63y) (period, 6 weeks)	Reductions of BW, BMI, TAG, and TNF-α levels, and increase of adiponectin	[172]
*Undaria pinnatifida*	Unknown	Meat containing 70 g ww of *U. pinnatifida* sporophyll	Interventional study in 12 healthy adults (average 25.4y) (period, 180 min)	Reductions of plasma glucose and its AUC	[173]
*Undaria pinnatifida*	Unknown	4 g dw of *U. pinnatifida* (FUERU WAKAME-CHAN^®^) with rice	An open-label, two-period, placebo-controlled, randomized trial in 26 healthy adults (average 51.5 y) (period, 120 min)	Reductions of blood glucose and insulin levels, and those AUC	[174]
*Undaria pinnatifida*	Unknown/d	A capsule containing 4 g dw of *U. pinnatifida* sporophyll/d for 4 weeks plus 6 g the alga/d for 4 weeks	Double-blind, placebo-controlled, randomized trial in 27 adults (average 46.2 y) with at least one symptom of the metabolic syndrome (period, 8 weeks)	Reductions of systolic blood pressure and waist circumference	[175]
*Undaria pinnatifida*	Unknown/d	A snack containing 32 mg dw of *U. pinnatifida*	Double-blind, placebo-controlled, randomized trial in 32 adults (average 51.1 y) with obese (period, 8 weeks)	Reductions of LDL-cholesterol, total-cholesterol and resistin level	[176]
*Saccharina japonica*	Unknown/d	1.5 g dw of fermented *S. japonica*	Double-blind, placebo-controlled, randomized trial in 48 healthy volunteers (period, 4 weeks)	Reductions of serum γ-GT and MDA, increases of SOD and CAT activities	[177]
*Saccharina japonica*	Unknown/d	6 g dw of roasted *S. japonica*	Interventional study in 52 adults (39–86 y) with normal and abnormal serum TAG levels (period, 4 weeks)	Reduction of serum TAG, Improvements of molecular species of PC, PE, LPC, LPE and FFA in the subjects with abnormal serum TAG level	[178,179]
*Saccharina japonica*	Unknown/d	A capsule containing 2.0 g dw of *S. japonica*	Double-blind, placebo-controlled, randomized trial in 70 healthy adults (average 56.6 y) (period, 6 weeks)	Reduction of body fat and improvement of adiponectin level	[180]
Unknown	2.4–8.0 mg/d	A capsule of 100 mg algal lipid-rich extract	Double-blind, placebo-controlled, randomized trial in 41 women with non-diabetic obese and NAFLD (period, 16 weeks)	Increase of resting energy expenditure	[166]
*Undaria pinnatifida* and *Saccharina japonica*	3.0 mg/d	A capsule containing 1.5 mg Fx powder (Fucoxanthin-P1^®^)	Double-blind, placebo-controlled, randomized trial in 33 adults (average 42.8 y) with a BMI ≥25 (period, 4 weeks)	Reductions of BMI, fat area, waist circumference	[32]

Fx, fucoxanthin; Dw, dry weight; BW, body weight; TAG, triacylglycerol; HOMA, homeostatic model assessment; BMI, body mass index; AUC, Area under the curve; ww, wet weight; LDL, lipoprotein; γ-GT, γ-glutamyltransferase; MDA, malondialdehyde; SOD, superoxide dismutase; CAT, catalase; NAFLD, non-alcoholic fatty liver disease.
